# Management of Isolated Axillary Recurrence in Breast Cancer: Is There a Role for Targeted Axillary Dissection?

**DOI:** 10.7759/cureus.88753

**Published:** 2025-07-25

**Authors:** Christina M Paluskievicz, Mahtab Vasigh, Piyush Rath, Seoho Lee, Lisa Jacobs, Melissa Camp, Julie Lange, David Euhus, Pam Wright, Bonnie Sun, Hanh-Tam Tran, Maureen O'Donnell, Olutayo Sogunro

**Affiliations:** 1 Surgical Oncology, Johns Hopkins University School of Medicine, Baltimore, USA; 2 Krieger School of Arts and Sciences, Johns Hopkins University, Baltimore, USA; 3 Surgery, Johns Hopkins University School of Medicine, Baltimore, USA

**Keywords:** axilla, axillary lymph node dissection, breast cancer, isolated axillary recurrence, survival, targeted axillary dissection

## Abstract

Background

The most optimal surgical strategy for ipsilateral isolated axillary recurrence (AR) in breast cancer is unknown. Axillary lymph node dissection (ALND) has historically been implemented; however, there may be an evolving role for targeted axillary dissection (TAD).

Methods

A retrospective analysis was conducted on patients with invasive breast cancer, followed by AR. Clinical and operative strategies were collected, with primary endpoints including overall survival (OS) and progression-free survival (PFS).

Results

In total, 21 (10.2%) patients were identified with AR. Of this subset of patients, 15 (71.4%) underwent ALND, and six (28.6%) underwent TAD. Univariable and multivariable analyses did not observe a significant association between TAD versus ALND at the time of recurrence with PFS. Univariable analysis demonstrated a significant association between adjuvant radiation and endocrine therapy at first recurrence and PFS benefit.

Conclusions

Following AR, no PFS benefit was observed between those managed with TAD versus ALND, which highlights an additional area of consideration for possible axillary de-escalation.

## Introduction

Axillary lymph node dissection (ALND) has previously been used as a standard means of locoregional control in breast cancer patients presenting with known axillary nodal metastases. Considering the significant morbidity associated with ALND and waning evidence of improvement in overall survival (OS), there has been movement away from the procedure to less invasive measures, such as lymphatic mapping and sentinel lymph node biopsy (SLNB), when appropriate [1-3]. Similarly, targeted axillary dissection (TAD) is a technique used following the completion of neoadjuvant systemic therapy in patients with known pathologically involved axillary nodes demonstrating an appropriate treatment response, to avoid ALND [4,5]. The technique is described as the removal of a previously clipped pathologic lymph node (LN), in combination with an SLNB using dual mapping, which results in an overall reduction in false negative rate for axillary staging to 2.0% after neoadjuvant chemotherapy [6,7].

Isolated axillary nodal recurrences (ARs) following an invasive breast cancer diagnosis are infrequent events, with rates described as ranging from 1% to 2%, but as high as 15%, likely as a result of significant advances in adjuvant systemic therapy [8]. AR most frequently occurs within 24-48 months following an initial breast cancer diagnosis [8]. Patients with isolated locoregional recurrences are at significant risk of distant recurrence, with five-year survival probability ranging from 45% to 80%, marking the necessity for adequate regional control strategies [9]. Potential factors playing a role in the development of AR include younger patient age, larger initial tumor volume, peritumoral vascular invasion, multicentricity or multifocality, non-luminal A subtype, and lack of treatment with radiotherapy [10,11]. Treatment for isolated AR disease has historically involved a multimodal approach, including isolated removal of involved LN or ALND, axillary radiation therapy, chemotherapy, and endocrine therapy [12]. Due to its rare occurrence, little data are available guiding the optimal surgical axillary strategy and the potential role of TAD as a therapeutic option in conjunction with systemic therapy. Therefore, we hypothesize that the use of ALND in patients presenting with AR may demonstrate no association with a progression-free survival (PFS) benefit compared to those treated with novel TAD.

## Materials and methods

Subjects

This was a retrospective study using a prospectively collected database of patients treated for breast cancer within the Johns Hopkins Hospital system. Patients greater than 18 years of age, with an invasive breast cancer diagnosis and a subsequent biopsy-proven recurrence in the ipsilateral axilla between September 2003 and September 2023, were analyzed using the database and electronic medical record. All patients included were treated within the Johns Hopkins Hospital system. This study was performed in accordance with Institutional Review Board approval (protocol IRB00414365). Due to the retrospective and non-interventional nature of this study, no written informed consent was required by the Institutional Review Board.

Patient characteristics assessed at the time of primary diagnosis include age at diagnosis, gender, race, and tumor laterality. Pathologic features of the primary tumor, including final histologic diagnosis, estrogen receptor status (ER), progesterone receptor status (PR), human epidermal growth factor receptor 2 (HER2) status, Ki-67%, tumor size, tumor grade, and original TNM (tumor, node, metastasis) status, according to the guidelines of the American Joint Committee on Cancer, were recorded. Recurrence data were recorded, including recurrence date, time to recurrence, recurrence location, TNM staging, and total number of recurrences. Surgical management of both the breast and axilla was determined based on review of dictated operative records at the time of initial diagnosis and following recurrence. Treatment modalities, including chemotherapy, radiation therapy, endocrine therapy, and HER2-targeted therapy, were recorded for both initial diagnosis and recurrent disease management. The primary endpoints include OS and analysis of PFS in the AR cohort [13].

Statistical analysis

Patient characteristics were summarized for the recurrence site using N (%) for categorical variables and mean ± SD for continuous variables. Differences between patient cohorts were tested using the Chi-square or Fisher’s exact test for categorical variables, and the t-test for continuous variables. OS was defined as the time from diagnosis to death. Patients who did not die were censored at the date of last follow-up. OS was estimated using the Kaplan-Meier method, and the log-rank test was used to test for differences in OS. Statistical significance was associated with a p-value < 0.05. We summarized patient characteristics, primary tumor characteristics, recurrent tumor characteristics, primary tumor management, and recurrent tumor management using N (%) for categorical variables and mean ± SD for continuous variables in the AR group. Time to first recurrence was calculated from the time of diagnosis to the time of first recurrence. Time to progression was calculated from the time of surgery for AR to the time of disease progression. Univariable and multivariable Cox regression models were created to determine factors associated with disease progression. All statistical analyses were conducted with IBM SPSS Statistics for Windows, Version 29 (Released 2021; IBM Corp., Armonk, NY, USA).

## Results

Baseline patient characteristics

**Figure 1:**
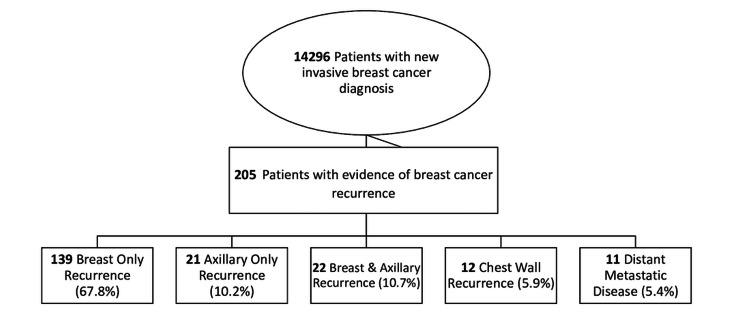
Study Flow Diagram

From September 2003 to September 2023, 14,296 patients were identified with a new invasive breast cancer diagnosis. Of this invasive cohort, 205 patients were found to have eventual histologic evidence of a breast cancer recurrence, with complete retrospective data available for analysis. A total of 139 (67.8%) patients were identified with breast-only recurrence, 21 (10.2%) with axillary-only recurrence, 22 (10.7%) with breast and AR, 12 (5.9%) with chest wall recurrence, and 11 (5.4%) with distant metastatic disease. A study flow design is pictured in Figure 1. Patient and tumor characteristics, stratified by recurrence location, are presented in Table 1.

**Table 1:** Characteristics of Tumors by Recurrence Location *Statistical significance was associated with a p-value < 0.05. Recurrence site was noted as N (%) for categorical variables and mean ± standard deviation for continuous variables. Differences between patient cohorts were tested using the Chi-square or Fisher’s exact test for categorical variables, and the t-test for continuous variables. ALND, Axillary lymph node dissection; TAD, Targeted axillary lymph node dissection; SLNB, Sentinel lymph node biopsy; HER2, Human epidermal growth factor receptor 2

Variable		Breast, n (%)	Axillary, n (%)	Breast and Axillary, n (%)	Chest Wall, n (%)	Distant, n (%)	Total, n (%)	p-value
		139 (67.8)	21 (10.2)	22 (10.7)	12 (5.9)	11 (5.4)	205	-
Sex	Female	139 (100)	19 (90.5)	22 (100)	11 (91.7)	11 (100)	202 (98.5)	0.003*
	Male	0	2 (9.5)	0	1 (8.3)	0	3 (1.5)	
Age		57.2 ± 15.4	54.9 ± 13.8	54.9 ± 15.2	52.8 ± 17.0	42.1 ± 12.9	15.4	-
Race	White	95 (68.3)	13 (61.9)	8 (36.4)	8 (66.7)	7 (63.6)	131 (63.9)	0.002*
	African American	33 (23.7)	2 (9.5)	10 (45.5)	3 (25.0)	4 (36.4)	52 (25.4)	
	Hispanic	4 (2.9)	0	0	0	0	4 (2.0)	
	Asian	2 (1.4)	2 (9.5)	2 (9.1)	1 (8.3)	0	7 (3.4)	
	Other	1 (0.7)	0	2 (9.1)	0	0	3 (1.5)	
	Unknown	4 (2.9)	4 (19.0)	0	0	0	8 (3.9)	
Histology	Invasive ductal carcinoma	108 (77.7)	21 (100)	20 (90.9)	11 (91.7)	10 (90.9)	170 (82.9)	0.69
	Invasive lobular carcinoma	12 (8.6)	0	0	1 (8.3)	0	13 (6.3)	
	Invasive mammary cancer	11 (7.9)	0	2 (9.1)	0	1 (9.1)	14 (6.8)	
	Other	3 (2.2)	0	0	0	0	3 (1.5)	
	Unknown	5 (3.6)	0	0	0	0	5 (2.4)	
Laterality	Left	68 (48.9)	13 (61.9)	8 (40.9)	8 (66.7)	4 (36.4)	102 (49.8)	0.75
	Right	69 (49.6)	8 (38.1)	13 (59.1)	4 (33.3)	7 (63.6)	101 (49.3)	
	Bilateral	2 (1.4)	0	0	0	0	2 (1.0)	
Estrogen receptor	Positive	97 (69.8)	20 (95.2)	13 (59.1)	9 (75.0)	8 (72.7)	147 (71.7)	0.02*
	Negative	25 (18.0)	1 (4.8)	9 (40.9)	3 (25.0)	3 (27.3)	41 (20.0)	
	Unknown	17 (12.2)	0	0	0	0	17 (8.3)	
Progesterone receptor	Positive	85 (61.2)	15 (71.4)	10 (45.5)	8 (66.7)	7 (63.6)	125 (61.0)	0.04*
	Negative	35 (25.2)	6 (28.6)	12 (54.5)	4 (33.3)	4 (36.4)	61 (29.8)	
	Unknown	19 (13.7)	0	0	0	0	19 (9.3)	
HER2	Negative	94 (67.6)	15 (71.4)	17 (77.3)	11 (91.7)	9 (81.8)	146 (71.2)	0.12
	Positive	17 (12.2)	6 (28.6)	4 (18.2)	1 (8.3)	0	28 (13.7)	
	Equivocal	5 (3.6)	0	1 (4.5)	0	0	6 (2.9)	
	Unknown	23 (16.5)	0	0	0	2 (18.2)	25 (12.2)	
Grade	1	18 (12.9)	0	0	3 (25.0)	0	21 (10.2)	<0.001*
	2	62 (44.6)	14 (66.7)	5 (22.7)	4 (33.3)	4 (36.4)	89 (43.4)	
	3	28 (20.1)	7 (33.3)	16 (72.7)	5 (41.7)	4 (45.5)	61 (29.8)	
	Unknown	31 (22.3)	0	1 (4.5)	0	2 (18.2)	34 (16.6)	
Size (cm)		1.7 ± 1.2	2.3 ± 1.7	2.0 ± 1.3	2.4 ± 1.2	1.9 ± 0.5	1.9 ± 1.3	-
pT	0	1 (0.7)	0	0	0	0	1 (0.5)	0.23
	1	86 (61.9)	14 (66.7)	13 (59.1)	6 (50.0)	7 (63.6)	126 (61.5)	
	2	31 (22.3)	4 (19.0)	8 (36.4)	5 (41.7)	3 (27.3)	51 (24.9)	
	3	3 (2.2)	2 (9.5)	1 (4.5)	1 (8.3)	0	7 (3.4)	
	4	0	1 (4.8)	0	0	0	1 (0.5)	
	Unknown	18 (12.9)	0	0	0	1 (9.1)	19 (9.3)	
pN	0	104 (74.8)	17 (81.0)	18 (81.8)	9 (75.0)	8 (72.7)	156 (76.1)	0.39
	1	16 (11.5)	4 (19.0)	4 (18.2)	3 (25.0)	3 (27.3)	30 (14.6)	
	Unknown or 2?	18 (12.9)	0	0	0	0	18 (8.8)	
Index breast surgery	Lumpectomy	133 (95.7)	13 (61.9)	17 (77.3)	5 (41.7)	4 (36.4)	172 (83.9)	<0.001*
	Mastectomy	5 (3.6)	4 (19.0)	4 (18.2)	3 (25.0)	4 (36.4)	20 (9.8)	
	Modified radical mastectomy	0	1 (4.8)	1 (4.5)	1 (8.3)	1 (9.1)	4 (1.8)	
	Nipple sparing mastectomy	0	2 (9.5)	0	3 (25.0)	1 (9.1)	6 (3.0)	
	Skin sparing mastectomy	0	1 (4.8)	0	0	1 (9.1)	2 (1.0)	
	Chest wall resection	1 (0.7)	0	0	0	0	1 (0.5)	
Index axillary surgery	SLNB	90 (64.7)	19 (90.5)	16 (72.7)	7 (58.3)	8 (72.7)	140 (68.3)	0.38
	ALND	31 (22.3)	1 (4.8)	4 (18.2)	4 (33.3)	3 (27.3)	43 (21.0)	
	None	18 (12.9)	1 (4.8)	2 (9.1)	1 (8.3)	0	22 (10.7)	
Death	Alive	109 (78.4)	20 (95.2)	14 (63.6)	7 (58.3)	8 (72.7)	158 (77.1)	0.2
	Dead	25 (18.0)	1 (4.8)	7 (31.8)	5 (41.7)	3 (37.3)	41 (20.0)	
	Unknown	5 (3.6)	0	1 (4.5)	0	0	6 (2.9)	

**Figure 2:**
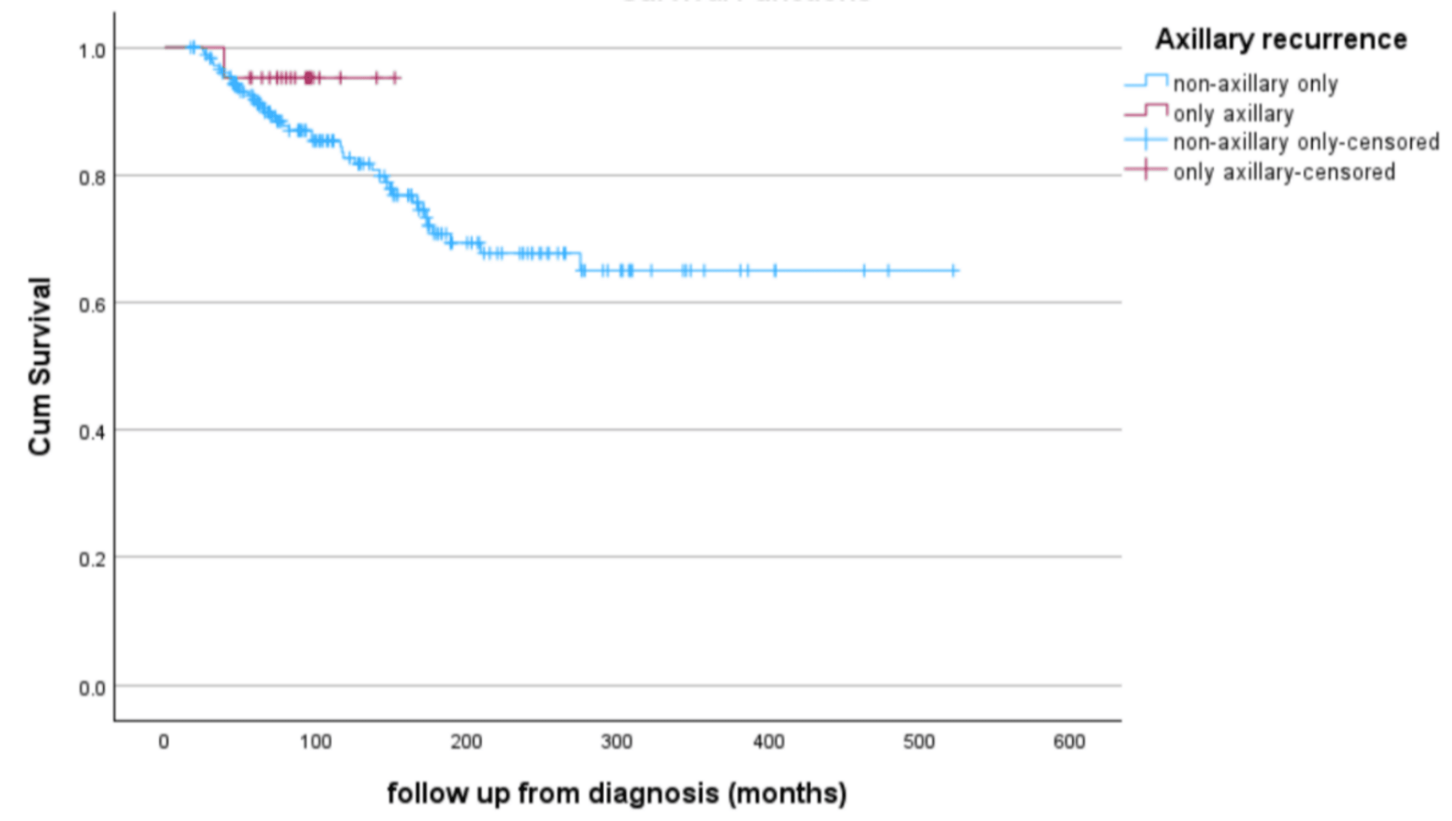
Overall Survival Comparing Axillary and Non-axillary Recurrences From the Time of Diagnosis Overall survival (OS) was defined as the time from diagnosis to death. If not deceased, patients were censored at the date of the last known follow-up. OS was estimated using the Kaplan-Meier method, and the log-rank test was used to test for differences in OS (p = 0.29).

Patients with distant metastatic disease at recurrence were excluded from further statistical analysis. There were significant differences between the groups with respect to patient gender (p = 0.006), race (p < 0.01), tumor grade (p < 0.001), original tumor size (p = 0.028), and type of surgery (p < 0.001). OS of isolated AR, in comparison to all other sites of disease recurrence - including breast only, breast and axilla, and chest wall - is displayed in Figure 2.

The survival rate of the entire cohort was 79.8% over a mean follow-up time of 48.8 months. The AR group had an OS of 95.2% during a follow-up time of 87.7 ± 26.5 months, and the other recurrent patients had an OS of 78.0% during a follow-up time of 102.8 ± 102.8 months (p = 0.06).

There was a mean survival time of 146.6 months (95% CI: 136.3-156.9) for patients with isolated AR, and a mean survival of 383.3 months (95% CI: 345.8-420.3) for all other recurrences. On Cox regression analysis, we did not observe any significant differences between patients with isolated AR and those with other recurrence types (OR: 0.35, 95% CI: 0.05-2.6; p = 0.3).

Isolated AR

Patient and disease characteristics for those with evidence of isolated AR are presented in Table 2. Of the patients identified, 19 (90.5%) were female, 13 (61.9%) were White, 21 (100%) had invasive ductal histology, and 20 (95.2%) and 15 (71.4%) had ER- and PR-positive lesions, respectively. The mean age at initial diagnosis was 53.2 ± 13.8 years, with a mean follow-up time from initial diagnosis of 87.7 ± 26.5 months (7.3 years). The mean time from diagnosis to first recurrence was 33.7 ± 22.4 months (2.8 years). At the time of isolated AR, 15 (71.4%) patients underwent ALND, six (28.6%) underwent TAD, and none underwent SLNB as a recurrent surgical management strategy. The mean follow-up time after the first recurrence was 23.1 months, and 28.6% experienced further disease progression, with one (4.8%) death observed in the cohort. Following histologic confirmation of regional disease recurrence, one (4.8%) patient was treated with neoadjuvant chemotherapy, and 13 (61.9%) were treated with adjuvant chemotherapy. Adjuvant radiation therapy was employed for 16 (76.2%) patients, and endocrine therapy was used for 15 (71.4%) patients.

**Table 2:** Characteristics of Axillary Recurrent Tumors We summarized patient characteristics, primary tumor characteristics, recurrent tumor characteristics, primary tumor management, and recurrent tumor management using N (%) for categorical variables and mean ± SD for continuous variables in the axillary recurrence (AR) group. ALND, Axillary lymph node dissection; TAD, Targeted axillary lymph node dissection; SLNB, Sentinel lymph node biopsy; HER2, Human epidermal growth factor receptor 2

Variable		Mean ± SD	Number	%
Patient Characteristics				
Sex	Female	-	19	90.5
	Male		2	9.5
Age at diagnosis		53.2 ± 13.8	-	-
Size of the tumor (cm)		2.3 ± 1.7	-	-
Time to first recurrence (months)		33.7 ± 22.4	-	-
Disease-free after the first recurrence		41.4 + 23.0	-	-
Race	White	-	13	61.9
	African American		2	9.5
	Asian		2	9.5
	Unknown		4	19
Primary Tumor Characteristics				
Histology	Invasive ductal carcinoma	-	21	100
Laterality	Left	-	14	66.7
	Right		7	33.3
Estrogen receptor	Positive	-	20	95.2
	Negative		1	4.8
Progesterone receptor	Positive	-	15	71.4
	Negative		6	28.6
HER2	Negative	-	15	71.4
	Positive		6	28.6
Grade	1	-	0	0
	2		14	66.7
	3		7	33.3
pT	1	-	14	66.7
	2		4	19
	3		2	9.5
	4		1	4.8
In breast primary surgical management	Lumpectomy	-	13	61.9
	Mastectomy		5	23.8
	Nipple sparing mastectomy		2	9.5
	Skin sparing mastectomy		1	4.8
Primary axillary surgery management	SLNB	-	19	90.5
	ALND		1	4.8
	None		1	4.8
Chemotherapy	Neoadjuvant	-	3	14.3
	Adjuvant		7	33.3
	None		11	52.4
Endocrine therapy for primary	Complete therapy course	-	13	61.9
	Incomplete therapy course		3	23.8
	Did not receive		5	14.3
Radiotherapy for primary	Received	-	10	47.6
	Did not receive		11	52.4
Recurrence Management				
Type of axillary surgery at recurrence	SLNB	-	0	0
	TAD		6	28.6
	ALND		15	71.4
Chemotherapy for recurrence	Neoadjuvant	-	1	4.8
	Adjuvant		13	61.9
	Did not receive		7	33.7
Radiotherapy for recurrence	Received	-	16	76.2
	Did not receive		5	23.8
Endocrine therapy for recurrence	Received	-	15	71.4
	Did not receive		6	28.6
Progression after the first recurrence	Progressed	-	6	28.6
	Did not progress		15	71.4
Death	Alive	-	20	95.2
	Died		1	4.8
Follow-up from primary diagnosis (months)	-	87.7 ± 26.5	-	-
Follow-up from first recurrence (months)	-	53.6 ± 23.1	-	-

AR management and PFS

**Figure 3:**
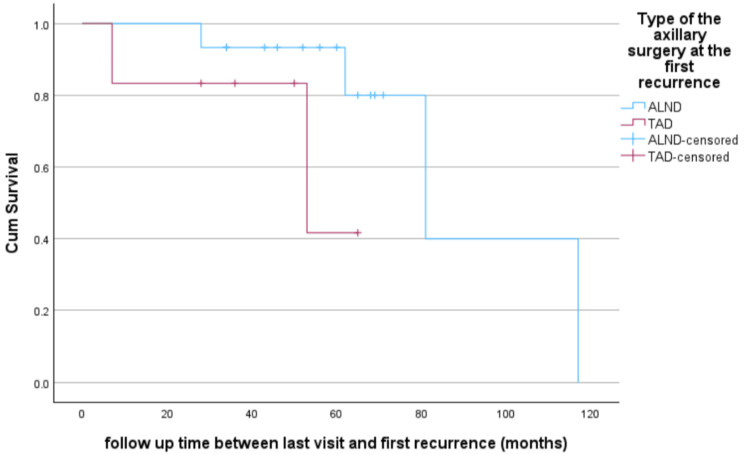
Progression-Free Survival After the First Axillary Recurrence Surgery Time to progression was calculated from the time of first recurrence to the time of disease progression. If no progression was documented, patients were censored at the date of the last known follow-up (p = 0.15). ALND, Axillary lymph node dissection; TAD, Targeted axillary lymph node dissection

We observed one death among 21 AR patients over a mean follow-up time of 53.5 ± 23.1 months, with a survival rate of 95.2%. Further disease progression (local, regional, or distant) was recorded in six patients (28.6%). The time to progression was calculated from the time of first recurrence to the noted progression. PFS for patients with isolated AR managed with TAD versus ALND is displayed in Figure 3.

We performed a univariable Cox regression model to determine factors associated with PFS. It was found that tumor size, grade, type of axillary or breast surgery at diagnosis, systemic treatment at the time of diagnosis or after the first recurrence, and time to first recurrence were not associated with PFS. In addition, we did not observe an association between axillary management (TAD vs. ALND) at the time of recurrence and PFS (OR: 1.34, 95% CI: 0.2-7.5; p-value = 0.7). However, there was a significant association between radiation treatment after the first recurrence and PFS (OR: 0.11, 95% CI: 0.02-0.6; p-value = 0.01). Similarly, treatment with endocrine therapy at recurrence was associated with a decreased probability of future disease progression (OR: 0.14, 95% CI: 0.03-0.79; p-value = 0.03). Multivariable analysis, adjusted for endocrine therapy, radiation treatment for the recurrence, primary tumor size, and endocrine therapy at diagnosis, did not reveal any significant associations between the type of axillary management at recurrence (TAD vs. ALND) and PFS (OR: 3.1, 95% CI: 0.2-60.3; p-value = 0.5). Table 3 displays univariable and multivariable analyses of predictors of PFS.

**Table 3:** Univariable and Multivariable Analysis of Progression-Free Survival After First Recurrence Based on Clinical Characteristics and Management in Patients With Axillary Recurrence *Statistical significance was associated with a p-value < 0.05. Univariable and multivariable Cox regression models were created to determine the factors associated with disease progression. ALND, Axillary lymph node dissection; TAD, Targeted axillary lymph node dissection; HER2, Human epidermal growth factor receptor 2

Univariable Analysis	OR	95.0% CI		p
		Lower	Upper	
Original tumor size (cm)	1.34	0.95	1.89	0.1
Grade at diagnosis	0.67	0.3	1.5	0.34
Estrogen receptor (ER) positivity at diagnosis	0.21	0	1315.5	0.73
Progesterone receptor (PR) positivity at diagnosis	1.12	0.48	2.62	0.79
HER2 positivity at diagnosis	0.91	0.35	1.9	0.63
Ki67 percentage at diagnosis	1	0.96	1.05	0.87
Type of Axillary Surgery at Diagnosis Compared to SLNB				
No axillary surgery at diagnosis	8.28	0	35776	0.62
ALND at diagnosis	0.35	0	189197	0.88
Type of Breast Surgery Compared to Lumpectomy at Diagnosis				
Mastectomy	10.83	0	-	0.98
Nipple sparing mastectomy (NSM)	5.75	0	-	0.98
Skin sparing mastectomy (SSM)	0	0	-	0.97
Administration of radiotherapy at initial diagnosis	0.91	0.41	2.04	0.83
Systemic Therapy at Initial Diagnosis Compared to No Treatment				
Adjuvant	0.82	0.27	2.47	0.73
Neoadjuvant	0.46	0.11	1.95	0.29
Endocrine therapy at diagnosis	1.89	0.84	4.22	0.12
Time to first recurrence	1	0.97	1.04	0.91
TAD at recurrence compared to ALND	1.34	0.24	7.5	0.74
Radiotherapy at recurrence	0.11	0.02	0.6	0.01*
Systemic therapy at recurrence	1.34	0.6	2.98	0.48
Endocrine therapy at recurrence	0.14	0.03	0.79	0.03*
Multivariable Analysis	OR	95.0% CI		p
		Lower	Upper	
Endocrine therapy at diagnosis	1.864	0.399	8.711	0.429
Endocrine therapy at recurrence	0.755	0.034	16.533	0.858
Radiotherapy at recurrence	0.094	0.006	1.607	0.103
TAD at recurrence compared to ALND	3.116	0.161	60.323	0.452
Original tumor size (cm)	0.827	0.491	1.392	0.474

## Discussion

Axillary management strategy remains an evolving topic in the surgical therapy of breast cancer. There is limited literature guiding the management of isolated AR following an invasive breast cancer diagnosis. Historically, surgical treatment necessitated ALND, which confers appreciable sequelae, including lymphedema, neurovascular compromise, and upper extremity mobility impairment [14]. The implementation of TAD, including SLNB with removal of a pathological node, is a less invasive, novel strategy that may achieve a similar degree of regional control within the axilla.

In our retrospective study of patients with invasive breast cancer who developed isolated AR, we demonstrated that surgical management using TAD is a suitable surgical strategy to achieve locoregional control, as an alternative to formal ALND. In our population, we did not observe a difference in PFS between the two groups - those who underwent TAD versus ALND. However, the data demonstrated that treatment with adjuvant radiation and endocrine therapy following regional recurrence was significantly associated with a PFS benefit, speaking to the critical role of adjuvant therapies and the importance of a multidisciplinary approach to breast cancer management.

There is currently limited literature specifically addressing our research question at hand for review; however, with the limited data available, some extrapolations can be made about our study population. de Boer et al. identify the rare occurrence of AR with a total cohort of 59 patients, and an overall actuarial survival rate of 39%, and a distant recurrence-free survival rate of 35% [15]. The study highlights the significant morbidity associated with full axillary clearance using ALND and speaks to the evolving role of axillary de-escalation in this patient population. Konkin et al. address the scant outcomes data related to management strategies in the setting of AR. Of 220 cases with identified AR, most patients underwent further axillary surgery (73.2%), including ALND (35%) and selective LN removal (65%), with no observed difference in OS [12]. Regional nodal radiation therapy was implemented at the time of isolated AR for nearly all patients when it was not offered as part of the primary adjuvant treatment plan, along with systemic therapy, including hormonal therapy, with noted improvement in disease-free survival and OS. This finding is echoed by Wright et al., who described a 10-year OS of 56% following AR and demonstrated that long-term survival is feasible if the disease is amenable to surgical intervention and aggressive adjuvant treatment [16]. Newman et al. aimed to characterize outcomes and management strategies of those with documented AR. A total of 44 patients with locoregional recurrence were identified, including 30 patients with isolated AR. Although techniques for surgical management are not specifically commented on, the authors do note that 75% of patients were managed with multimodal therapy, with the most common sequence of intervention being surgery followed by systemic therapy and/or nodal radiation [17]. Similar to Konkin et al. [12], we observed no difference in PFS when evaluating those managed with a more selective axillary approach versus ALND. Much like our findings, this described body of literature also draws attention to the critical role that systemic adjuvant therapies play in the management of this recurrence type.

It is not clear from Konkin et al. [12] what specific surgical technique is termed selective axillary surgery, and whether this refers to SLNB or TAD. This draws attention to the relevance and importance of our study. Unlike the previous research discussed, our study is novel in that we are posing a specific question - not only inquiring about patient clinical outcomes in the setting of AR, but also delving into the details of the surgical approach to the axilla. The importance of our study lies in its focus on the specific use of TAD, with no observed difference in PFS between the two groups, which could support further de-escalation of the axilla. Based on our limited retrospective evaluation, it appears that TAD has an emerging role over ALND in the management of AR with regard to disease outcomes.

The potential limitations of our study should be well noted. The study was retrospective, non-randomized in nature, which could potentially be influenced by confounding variables outside of our control. Secondly, with AR being a rare event, we were only able to identify a small sample size with complete data within a single institution, which speaks to the need for larger-scale, multi-institutional studies in the future. In addition, a randomized prospective trial focusing on AR management is a necessity in establishing guidelines of care for this patient population. However, it is understood that this will be highly challenging, considering the rare nature of the event in the population at hand, making enrollment a significant challenge. Lastly, our study did not classify the number of nodes involved at the time of recurrence or the size of tumor deposits focused within the involved node. Having this information would have provided the ability to stratify patients based on pathologic involvement and provided additional prognostic factors that can contribute to further disease progression. Knowing this, in a clinical setting, could ultimately assist in decision-making about the most appropriate situations to offer TAD versus ALND. Despite these limitations, this study provides eye-opening data that could potentially help guide the management of AR in the future.

## Conclusions

In conclusion, TAD has an evolving role in the management of patients with identified isolated AR as a potential alternative to formal ALND. Adequate treatment of AR should not only focus on local management measures but also on adjuvant radiation and systemic therapy options when appropriate. Further investigation, using larger patient cohorts and randomization, is required to ascertain the feasibility of TAD and its application in this population moving forward.
